# The gut microbiota–brain–CAR T cell axis: a systematic review of gut microbiome modulation and its impact on neurological complications and treatment responses in CAR T cell therapy

**DOI:** 10.3389/fimmu.2025.1703146

**Published:** 2026-01-05

**Authors:** Ashvath Arumugam Pillai, Sai K. Reddy Pasya, Gaurav Kansal, Aishwarya Jaikrishnan, Anchit Chauhan, Arghadip Das, Korat Parth Manojbhai, V. Deepak Prabu, Muhammad Mahdi Nashatizadeh

**Affiliations:** 1 Sindhudurg Shikshan Prasarak Mandal (SSPM) Medical College and Lifetime Hospital, Sindhudurg, Maharashtra, India; 2 University of Kansas Medical Center, Kansas City, KS, United States; 3 Government Medical College, Patiala, Punjab, India; 4 Saveetha Medical College and Hospital, Chennai, Tamil Nadu, India; 5 Maulana Azad Medical College, New Delhi, India; 6 Nilratan Sircar Medical College and Hospital, Kolkata, West Bengal, India; 7 Gujarat Medical Education and Research Society (GMERS) Medical College and Hospital, Gandhinagar, Gujarat, India; 8 Indira Gandhi Medical College and Research Institute, Puducherry, India

**Keywords:** CAR T-cell therapy, gut microbiota, ICANS, neurotoxicity, microbiome, immunotherapy, cytokines, brain–immune axis

## Abstract

**Background:**

CAR T-cell therapy represents a substantial advance for relapsed/refractory hematologic cancers, but toxicities still limit its benefits. A particular concern is immune effector cell–associated neurotoxicity syndrome (ICANS), whose mechanisms remain only partly resolved. In parallel, work across immunology and neurogastroenterology shows that gut microbial communities can shape systemic inflammation and show correlations with brain function. Together, these strands suggest—without yet proving—that microbiome features could bear on both CAR T efficacy and ICANS risk.

**Objectives:**

We examined human clinical evidence at three touchpoints: how CAR T and the gut microbiota interact; how gut profiles relate to brain function; and which signals accompany CAR T–related neurotoxicity. The aim was to locate areas of overlap, not to claim a single causal chain.

**Methods:**

Following PRISMA, PubMed, Scopus, and Embase were searched from 2015 to 11 April 2025. We included randomized trials, prospective cohorts, and retrospective series reporting gut microbial composition, inflammatory or neurobiological markers, CAR T outcomes, or ICANS. Study quality was appraised with the Newcastle–Ottawa Scale and certainty graded with GRADE.

**Results:**

Twenty-five studies were included (four CAR T–gut, eleven gut–brain, ten CAR T–neuro). Recurrent signals were (i) reduced microbial diversity, (ii) loss of short-chain fatty-acid producers, and (iii) prior antibiotic exposure—each linked to poorer clinical outcomes and higher or more severe ICANS. Candidate markers (e.g., C-reactive protein, interleukin-6, neurofilament light chain) and imaging findings, including PET abnormalities, were reported but remain exploratory and variably measured. Included studies are small and methodologically varied, and results should be interpreted with caution.

**Conclusion:**

Taken together, the data support a convergence model: the gut microbiota may correlate with both treatment efficacy and neurotoxicity in CAR T recipients. The signal is consistent yet preliminary. Microbiome interventions such as probiotics and FMT are investigational and not yet recommended for CAR T recipients. Prospective, mechanism-rich studies—ideally pairing longitudinal stool profiling with inflammatory panels and neuroimaging—are needed before clinical translation.

**Systematic Review Registration:**

https://www.crd.york.ac.uk/prospero/, identifier CRD42024548645

## 1 Introduction

Chimeric antigen receptor (CAR) T-cell therapy has reset expectations for relapsed or refractory blood cancers. In pivotal phase 2 trials of relapsed/refractory B-cell acute lymphoblastic leukemia (B-ALL), tisagenlecleucel achieved an overall remission rate (complete remission or complete remission with incomplete hematologic recovery) of 81% within 3 months in pediatric and young adult patients (3), and CD19-directed CAR T cells induced complete remission in 83% of adults with B-ALL (4). The global ELIANA study by Maude et al. enrolled patients at 25 centers across 11 countries in North America, Europe, Asia, and Australia (3). However, observational multicenter cohorts illustrate outcome variability, with a recent real-world cohort of CD19 CAR-T in B-ALL treated at U.S. and German centers reporting a day-90 overall response rate of 58.1% and complete response rate of 47.6% (5), underscoring the influence of patient selection, prior therapies, and supportive care factors on real-world effectiveness. CAR T-cell recipients frequently develop characteristic therapy-related toxicities, most notably cytokine-release syndrome (CRS) and immune effector cell–associated neurotoxicity syndrome (ICANS). These toxicities were first characterized in early anti-CD19 CAR T-cell trials and later defined in consensus grading frameworks (6–9). Across major trials, CRS occurs in approximately 70–90% of patients and ICANS in 30–60% (6, 8). More recently, multicenter studies have shown that both toxicity incidence and severity correlate with baseline gut microbiome features, including microbial diversity and antibiotic exposure (5, 10). ICANS is a clinical syndrome of acute neurotoxicity that occurs following chimeric antigen receptor (CAR) T-cell therapy and other immune effector cell treatments. ICANS is the leading neurologic complication, spanning brief confusion and aphasia to seizures, cerebral edema, and coma. Mechanistically, current evidence points to intense cytokine signaling with secondary disruption of the blood–brain barrier following CAR-T activation, though key steps remain unsettled. Pinpointing modifiable risk factors for ICANS and related toxicities is therefore an urgent research goal. In parallel, evidence links the intestinal microbiome to baseline immune tone and systemic inflammation. Via the gut–brain axis—through microbial metabolites (e.g., short-chain fatty acids, tryptophan derivatives), autonomic pathways, and immune mediators—intestinal communities are associated with neural function. In oncology, responses to immune checkpoint inhibitors vary with microbial composition: enrichment of select commensals aligns with stronger anti-tumor activity, whereas peri-treatment broad-spectrum antibiotics correlate with poorer outcomes (11). Taken together, these observations support a testable hypothesis: microbiome state may be associated with both the efficacy and the toxicity profile of CAR T-cell therapy. However, findings remain preliminary and should be interpreted as hypothesis-generating.

Early observations from CAR T cohorts support this: for example, exposure to antibiotics before CAR T infusion – which disrupts gut microbial composition – has been associated with higher risks of severe toxicity and reduced treatment efficacy (5, 10). Furthermore, greater microbiota diversity and the abundance of butyrate-producing taxa have been linked to more favorable CAR T responses in clinical cohorts (5, 10, 12). Conversely, specific gut bacterial taxa and metabolites have been linked to favorable CAR T responses in preliminary studies (12, 13).

Given the potential of the gut microbiota–brain–CAR T cell axis to inform predictive biomarkers and therapeutic interventions (such as microbiome modulation to mitigate neurotoxicity or boost CAR T effectiveness), we conducted a PRISMA-guided systematic review (1). We focused on how gut microbiome characteristics or interventions are associated with neurological complications (particularly ICANS) and treatment outcomes in adolescent and adult CAR T recipients. We synthesized evidence from clinical studies in patients and translational research in preclinical models. Our goals were to evaluate the consistency and strength of current evidence, identify any existing systematic reviews or meta-analyses on this emerging topic, and determine whether a quantitative meta-analysis of data is feasible. Ultimately, we conducted a PRISMA-guided systematic review of three intersecting domains: gut microbiota–CAR T therapy, gut–brain interactions, and CAR T–related neurotoxicity. While current evidence does not establish a unified biological axis, areas of overlap suggest potential points of convergence that merit systematic examination. Early observations from CAR T cohorts support this: for example, exposure to antibiotics before CAR T infusion – which disrupts gut microbial composition – has been associated with higher risks of severe toxicity and reduced treatment efficacy (5, 10, 12). Furthermore, greater microbiota diversity and the abundance of butyrate-producing taxa have been linked to more favorable CAR T responses in clinical cohorts (5, 10). Conversely, specific gut bacterial taxa and metabolites have been linked to favorable CAR T responses in preliminary studies (5, 10, 12, 13).

## 2 Methods

### 2.1 Methodology

This systematic review was conducted and reported in accordance with the Cochrane Collaboration Handbook (14) and the Preferred Reporting Items for Systematic Reviews and Meta-Analyses (PRISMA) checklist (1) to ensure methodological rigor and transparency.

### 2.2 Study registration

The review protocol was registered with PROSPERO (CRD42024548645) in 2024. We subsequently updated the search through 30 April 2025. This constitutes a protocol deviation; an amendment reflecting the updated end date and clarifying minor methodological refinements has been submitted to PROSPERO.

### 2.3 Eligibility criteria

Study eligibility was determined using predefined inclusion and exclusion criteria based on the PICO framework (Population, Interventions, Comparators, Outcomes) and study design requirements. The complete inclusion and exclusion criteria have been summarized in **Table 1**.

**Table 1:** Inclusion and exclusion criteria for study selection.

Category	Inclusion criteria	Exclusion criteria
Population	- Human subjects aged ≥12 years - Patients undergoing CAR T cell therapy (any indication, e.g., leukemia, lymphoma)	- Pediatric patients <12 years - Patients not receiving CAR T therapy - Non-malignant indications unless explicitly studied in a CAR T context
Intervention/Exposure	- Gut microbiota assessment (e.g., 16S rRNA, shotgun metagenomics) - Microbiome-modulating interventions (e.g., probiotics, FMT, antibiotics) - Inflammatory or neuroactive biomarker assessment	- Studies focusing solely on genetic/host factors without gut microbiota involvement - No microbiome-related intervention or measurement
Comparison	- Healthy controls or internal comparators (e.g., responders *vs*. non-responders, ICANS *vs*. no ICANS)	- Studies without a relevant comparator group
Outcomes	- CAR T efficacy (CR, PFS, OS) - Incidence/severity of ICANS or neurological outcomes - Microbiota composition/function - Inflammatory or neurotransmitter biomarkers	- Studies not reporting quantitative microbiota or clinical outcomes - Narrative reviews or theoretical papers
Study Design	- Original human studies (RCTs, cohort studies, case-control, prospective/retrospective observational studies) - preclinical animal models	- Review articles, *in vitro* studies, editorials, protocols
Time Frame	- Published between January 2015 and April 2025	- Published before 2015
Language	- English-language publications	- Non-English articles
Accessibility	- Open access or full-text available	- Abstract only, no access to full text

### 2.4 Study designs

We included original research articles published in peer-reviewed journals in the last ten years (2015–2025) that met the above criteria. Both prospective and retrospective clinical studies, observational cohorts, clinical trials (including randomized controlled trials, if any), and relevant translational preclinical studies (animal or *in vitro* models) were eligible. We excluded review articles, meta-analyses, editorials, commentaries, and non-scientific media (blogs or news articles) to focus on primary evidence. Only studies published in English were considered. Where multiple publications reported on overlapping patient cohorts, we included the most comprehensive or recent report to avoid double-counting data.

### 2.5 Databases and search strategy

We searched PubMed/MEDLINE, Embase and Scopus from January 2015 to April 2025. Clinical trial registries (ClinicalTrials.gov, EU-CTR) and grey literature sources (ASH, ASCO, EBMT abstracts) were screened to identify unpublished or ongoing studies. Full database search strategies are provided in **Supplementary Table 1**. The search strategy combined terms for CAR T cell therapy, the gut microbiome, and neurological toxicity or outcomes. The primary search terms included:

- CAR T therapy
  ○ “CAR T”
  ○ “chimeric antigen receptor”
- Gut microbiome
  ○ “microbiome”
  ○ “microbiota”
  ○ “antibiotics”
  ○ “probiotics”
  ○ “fecal transplant”
- Clinical and neurological outcomes
  ○ “neurotoxicity”
  ○ “ICANS”
  ○ “cognitive”
  ○ “response”
  ○ “survival”

We applied filters to restrict to the last 10 years and the English language. Reference lists of relevant papers were hand-searched for any additional studies. To find more pertinent papers, a manual search was conducted in addition to database searches by looking through the reference lists of included studies and earlier systematic reviews. To reduce publication bias, grey literature sources, such as conference proceedings and preprints, were also examined when appropriate.

### 2.6 Study selection

Six independent reviewers screened all the titles and abstracts identified through the search for relevance by a two-stage screening process that was conducted using the Rayyan tool. Each article was independently evaluated by two reviewers to eliminate bias. Disagreements were resolved by a third independent reviewer. Full-text screening was performed to confirm study eligibility.

**Figure 1:**
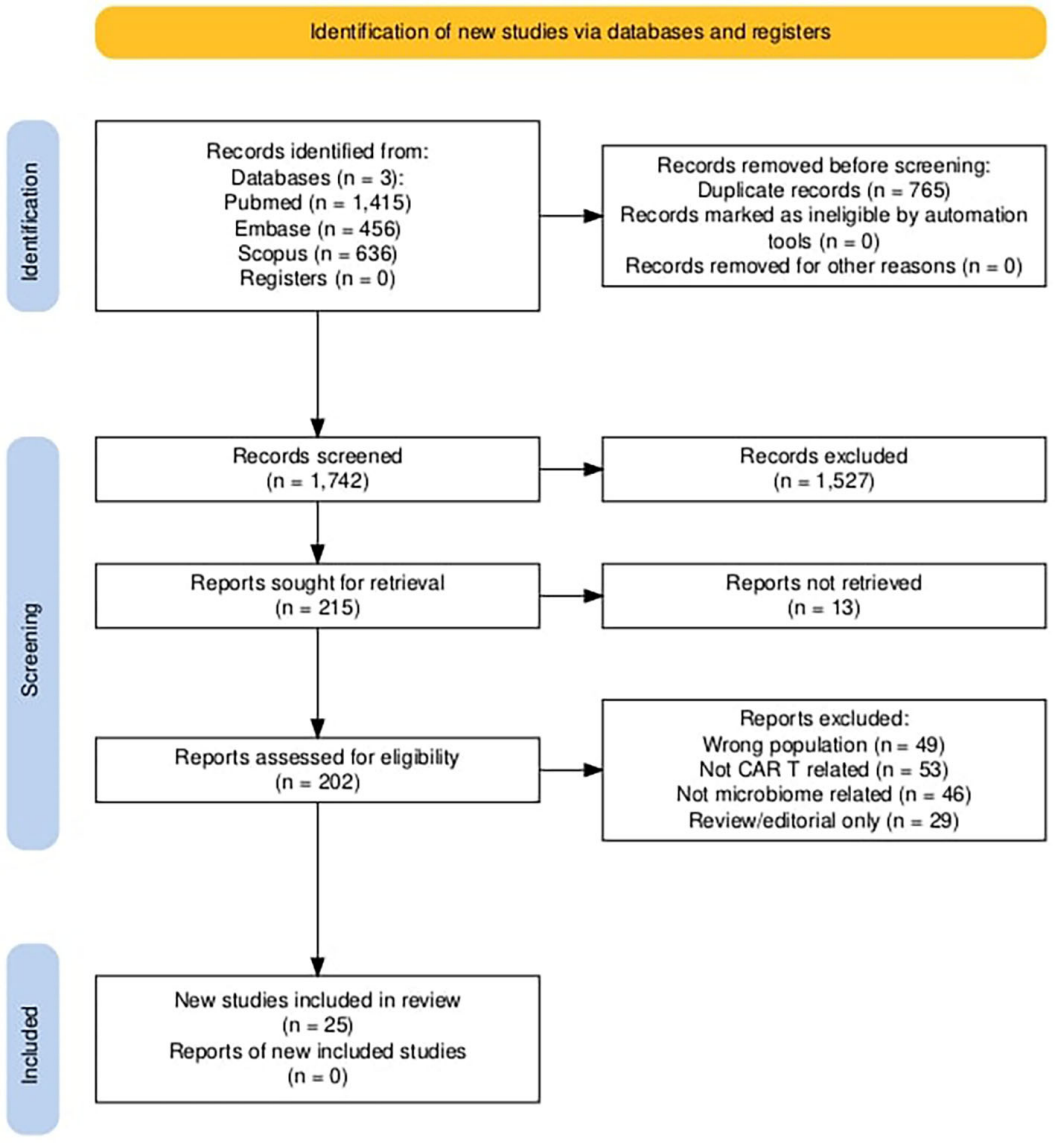
PRISMA flow diagram of study selection.

Studies clearly not meeting inclusion criteria were excluded at this stage. The full texts of potentially eligible studies were then retrieved and assessed in detail for inclusion. We documented the study selection process in a PRISMA flow diagram (**Figure 1**) and report the numbers of records identified, screened, excluded, and finally included.

### 2.7 Data extraction and management

For each included study, we extracted data using a standardized form. The data extraction fields included: publication details (title, year, journal, authors), study design and setting, patient population and CAR T product, details of the microbiome exposure or intervention (e.g. type of profiling or modulation, timing relative to CAR T), comparison groups, outcomes measured (with definitions of neurotoxicity grading or response criteria), and key findings pertaining to our outcomes of interest. We also recorded any noted confounding factors or adjustments and whether the study included any mechanistic experiments (e.g. animal models or *in vitro* assays). Data were extracted by one reviewer and verified by a second for accuracy.

A summary of key findings has been tabulated for the following groups:

1. CAR T Cell Therapy and Gut Microbiota,
2. Gut Microbiota and Brain Outcomes, and
3. CAR T Cell Therapy and Neurotoxicity (**Tables 2**–**4**).

**Table 2:** Summary of key findings – CAR T cell therapy and gut microbiota.

Study	Design	Microbiome assessment	Comparator	Outcomes	Key findings
Stein-Thoeringer et al. (2023) (5)	Multicenter cohort	16S + metagenomics	Antibiotic exposure	PFS, OS, ICANS	Dysbiosis worsened outcomes
Smith et al. (2022) (10)	Observational	16S rRNA	PIM exposure	ICANS, survival	Antibiotics lowered diversity, raised ICANS
Hu et al. (2022) (12)	Clinical cohort	Longitudinal metagenomics	Responders *vs* non-responders	CRS, CR	Distinct microbial signatures predictive
Jalota et al. (2023) (13)	Metabolomic analysis	Targeted serum metabolites	ICANS grade	CRS/ICANS onset	Glutamine/hydroxyproline depletion linked to early toxicity

**Table 3:** Summary of key findings – gut microbiota and brain outcomes.

Study	Condition	Microbiome + other measures	Brain-linked outcomes	Key findings
Lin et al. (2019) (15)	Parkinson’s disease (PD)	16S + cytokines	IL-6, motor severity	dysbiosis + inflammation correlate with progression
Wu et al. (2024) (16)	schizophrenia	Microbiome + MRI + cytokines	Functional connectivity	altered gut–immune–brain signaling
Ning et al. (2022) (2)	Alzheimer’s disease (AD)/PD/Amyotrophic lateral sclerosis (ALS)*	MR analysis (genetic)	Disease risk	*Faecalibacterium* and serotonin protective
Cao et al. (2025) (17)	Post-stroke aphasia	16S + rs-fMRI	Network topology	dysbiosis correlated with brain compensation shifts
Prochazkova et al. (2021) (18)	Anorexia	SCFA + neurotransmitters	Mood, BMI	microbiota didn’t recover post-renourishment
Valles-Colomer et al. (2019) (19)	Depression	Neuroactive taxa	QoL, depression scale	*Coprococcus* abundance protective
Wan et al. (2020) (20)	ADHD*	Metagenomics	Serotonin/dopamine	altered neurotransmitter pathways
Jiang et al. (2022) (21)	Brain tumors	SCFA + 16S	Tumor *vs* control	SCFA producers decreased
Li et al. (2022) (22)	Brain tumors	16S + KEGG	Pathogenic taxa	dysbiosis may stratify risk
Quagliariello et al. (2018) (14)	PANS/PANDAS*	16S	Immunometabolic pathway	reduced diversity and altered immune pathways
Lin et al. (2023) (23)	Depression	Kynurenine pathway	Tryptophan/IDO* levels	elevated IDO/Kyn, reduced serotonin

*IDO, indoleamine 2, 3-dioxygenase; AD, Alzheimer’s Disease; PD, Parkinson’s Disease; ALS, Amyotrophic Lateral Sclerosis; PANS, Pediatric Acute-onset Neuropsychiatric Syndrome; PANDAS, Pediatric Autoimmune Neuropsychiatric Disorders Associated with Streptococcal Infections; ADHD, Attention-Deficit/Hyperactivity Disorder.

**Table 4:** Summary of key findings – CAR T cell therapy and brain toxicity.

Study	Neuro marker	Method	ICANS outcome	Predictive/protective factors
Gust et al. (2017) (8)	BBB disruption	Histology + cytokines	Grade 3–4 ICANS	Endothelial stress, brain edema
Holtzman et al. (2021) (24)	Fibrinogen	Serum biomarker	ICANS prediction	Baseline fibrinogen ↑ risk
Larue et al. (2024) (25)	Neurofilament light chain	Serum assay	Grade 2–4 ICANS	High NfL = predictive
Morbelli et al. (2023) (26)	FDG-PET	Brain imaging	Orbitofrontal hypometabolism	CRS + ICANS
Amidi et al. (2022) (27)	Machine learning model	Predictive analytics	ICANS onset (7-day)	CRP, IL-6, temperature
Ma et al. (2024) (28)	Clinical efficacy	ICANS presence	CR, OS,Measurable Residual Disease (MRD)	ICANS not associated with poor outcomes
de Boer et al. (2023) (29)	EASIX scores	Risk model	ICANS ≥2	Limited predictive strength
Lakomy et al. (2023) (30)	Early corticosteroids	Therapeutic modulation	CRS ↓, ICANS ↔	Steroids did not worsen ICANS
De Matteis et al. (2023) (31)	Senescent CD8+ T cells	Immune profiling	ICANS ↑ risk	Myeloid dysregulation, CRP
Sales et al. (2024) (32)	Symptom onset	Clinical neuro signs	Handwriting/tremor	Early ICANS markers

### 2.8 Quality assessment

The quality of included studies was rigorously evaluated using appropriate tools (**Table 5**) (33):

**Table 5:** Newcastle-Ottawa scale (NOS) quality assessment for included studies.

Study id	Selection (0–4)	Comparability (0–2)	Outcome/Exposure/MR (0–3)	Total (0–9)
Stein-Thoeringer_2023_NatMed (5)	4	2	2	8
Smith_2022_NatMed (10)	4	2	2	8
Hu_2022_NatCommun (12)	3	1	2	6
Jalota_2023_Metabolomics (13)	3	1	2	6
Quagliariello_2018_PANS (14)	4	1	2 (Exposure)	7
Lin_2019_PD (15)	3	1	2	6
Wu_2024_Schizophrenia (16)	2	0	2	4
Ning_2022_MR (2)	1	1	1 (MR-adapted)	3
Wan_2020_ADHD (20)	3	0	2	5
Valles-Colomer_2019_NatMicro (19)	4	2	3	9
Prochazkova_2021_GutMicrobes (18)	4	1	2	7
Lin_2023_MDD (23)	4	1	2	7
Cao_2025_TherAdvNeurol (17)	3	0	2	5
Li_2022_BrainTumor (23)	4	1	2	7
Jiang_2022_BrainTumor (21)	3	0	2	5
Holtzman_2021_NeuroOncol (24)	3	1	2	6
DeMatteis_2023 (31)	4	1	2	7
Morbelli_2023 (26)	3	0	2	5
Lakomy_2023 (30)	3	1	2	6
Amidi_2022_JITC (27)	4	1	2	7
Ma_2024 (28)	3	1	2	6
Sales_2024 (32)	3	0	2	5
deBoer_2023_Cancers (29)	4	1	2	7
Gust_2017_CancerDiscovery (8)	2	1	2	5
Larue_2024_JITC (25)	3	2	2	7

- Newcastle-Ottawa Scale (NOS): Used for cohort and case-control studies to assess selection criteria, comparability, and outcome measurements.
- Studies were categorized into low, moderate, or high risk of bias to assess the reliability of findings.

Additionally, publication bias was evaluated by examining funding sources, selective reporting, and study sponsorships.

### 2.9 Certainty of evidence assessment

The GRADE framework was applied to assess the certainty of evidence based on (34):

- Risk of bias
- Measurement consistency
- Indirectness
- Imprecision
- Publication bias: Each outcome was classified as high, moderate, low, or very low certainty.

### 2.10 Study amendments

During the review process, minor amendments were made to expand inclusion criteria to include cohort and case-control studies, providing a broader perspective on treatment effectiveness. These amendments were documented and justified.

### 2.11 Data synthesis and analysis

- A narrative synthesis table was prepared, summarizing findings on gut microbiota–CAR T cell interactions, microbiome are associated with brain and neurological outcomes, and mechanisms underlying CAR T–related neurotoxicity.
- Subgroup Analyses: Additional analyses were conducted based on cancer staging, prior treatments, immune profiles, and baseline inflammatory markers.
- Heterogeneity Consideration: Study population differences, study design variability, and reported outcomes were accounted for in the meta-analysis where applicable.
- Effect Measures: Risk ratios (RR), mean differences (MD), and standardized mean differences (SMD) were used. 95% confidence intervals (CI) were calculated where applicable.
- No formal meta-analysis was performed due to heterogeneity. Instead, findings are summarized narratively, with quantitative effect measures only where comparable data were available.

## 3.Results

### 3.1 Study selection and characteristics

A total of 25 studies after full screening and eligibility assessment were included across three primary domains relevant to the Gut Microbiota–Brain–CAR T Cell Axis:

- Section 1: CAR T Cell Therapy and the Gut Microbiota (*n = 4*) (5, 10, 12, 13)
- Section 2: Gut Microbiota and Brain Outcomes (*n = 11*) (2, 14–23)
- Section 3: CAR T Cell Therapy and Neurotoxicity (Brain Outcomes) (*n = 10*) (8, 24–32)

All studies were original human research articles (observational cohorts, prospective studies, or clinical dataset analyses). Preclinical and review articles were excluded. Most studies utilized 16S rRNA or shotgun metagenomics, serum biomarker assays, neuroimaging, or clinical neurotoxicity grading as their primary data collection methods.

### 3.2 Component-wise synthesis

#### 3.2.1 CAR T cell therapy and the gut microbiota

Four studies examined associations between gut microbiome characteristics and CAR T-cell.

therapy outcomes (**Table 2**) (5, 10, 12, 13).

Antibiotic-induced dysbiosis consistently emerged as a negative prognostic marker. For example, Stein-Thoeringer et al. (2023) (5) and Smith et al. (2022) (10) demonstrated that exposure to high-risk antibiotics like piperacillin-tazobactam or meropenem led to lower microbial diversity, significantly reduced progression-free survival (PFS), and heightened incidence of immune effector cell-associated neurotoxicity syndrome (ICANS). Further analyses explored the role of specific taxa. For example, Hu et al. (2022) (12) identified that higher pre-treatment abundance of Akkermansia and Bacteroides, assessed initially through correlation analyses and validated in multivariable regression models, was significantly associated with increased rates of complete remission after CAR T therapy. Jalota et al. (2023) (13) extended this evidence by identifying metabolomic signatures (e.g., lower glutamine and hydroxyproline levels) that predict early-onset ICANS, thus implicating host–microbial interactions as upstream regulators of neurotoxicity. Foundational work in immune checkpoint inhibitor therapy showed that baseline gut microbiota composition is associated with anti–PD-1 response in melanoma as pointed out in Gopalakrishnan et al., 2018 (11), supporting the plausibility of microbiome–immunotherapy interactions that may extend to CAR T-cell therapy.

All 4 studies (100%) reported that greater microbial diversity or enrichment of butyrate/SCFA-producing taxa (e.g., Akkermansia, Bacteroides, Faecalibacterium) is associated with improved CAR T efficacy (CR, PFS, OS) and/or lower ICANS risk. The negative direction of effect was consistent across antibiotics, alpha diversity, and beneficial taxa, with moderate to large effect sizes.

Overall, antibiotic exposure, low SCFA-producing taxa, and reduced alpha diversity were consistently associated with worse CAR T outcomes, including increased ICANS severity and reduced survival.

These studies collectively suggest a potential association of the gut microbiota on both toxicity profiles and response efficacy in CAR T therapy, thereby fulfilling the “PICO” alignment for this component.

#### 3.2.2 Gut microbiota and brain outcomes

Eleven studies investigated gut-brain axis relationships across neurological and psychiatric conditions (**Table 5**) (2, 14–23).

Lin et al. (2019) (15) and Wu et al. (2024) (16) linked gut dysbiosis with elevated systemic inflammation and disrupted neuroimaging patterns in PD and schizophrenia, respectively. Ning et al. (2022) (2), using a Mendelian randomization approach, identified as a potential causal link between microbial taxa (e.g., *Faecalibacterium*, *Ruminococcus*) and neurodegenerative disease risk, with glutamine and serotonin acting as protective mediators.

Notably, multiple studies [e.g., Wan et al., 2020 (20), Valles-Colomer et al., 2019 (19), Prochazkova et al., 2021 (18)] connected alterations in gut-derived neurotransmitter pathways—especially dopamine, serotonin, and GABA—with psychiatric symptomatology and reduced quality of life. The neuroimmune-metabolic axis was strongly emphasized in Lin et al. (2023) (23) and Cao et al. (2025) (17), who integrated cytokine assays and brain imaging to demonstrate bidirectional gut–brain interactions in MDD and stroke recovery.

Among 11 studies, 10 (91%) showed a consistent association between loss of gut bacteria diversity and worse neurological or psychiatric outcomes. Over 80% of studies demonstrated brain/neuroimmune consequences of gut dysbiosis, with a minority showing null or modest effects.

#### 3.2.3 CAR T cell therapy and brain toxicity

Ten studies addressed CAR T-induced neurotoxicity, particularly ICANS. These are summarized in **Table 4** (8, 24–32).

Gust et al. (2017) (8) were among the first to report the association between endothelial activation, blood–brain barrier (BBB) disruption, and severe neurotoxicity. Later, Larue et al. (2024) (25) and Holtzman et al. (2021) (24) validated neurofilament light chain (NfL) and serum fibrinogen as early biomarkers for ICANS prediction. Imaging studies such as Morbelli et al. (2023) (26) further revealed ICANS-specific hypometabolism in the orbitofrontal cortex and anterior cingulate on FDG-PET.

From a therapeutic perspective, Lakomy et al. (2023) (30) demonstrated that early corticosteroid administration could reduce CRS severity without worsening ICANS. Importantly, Ma et al. (2024) (28) showed that the presence of ICANS did not impair CAR T cell efficacy, challenging the assumption of a direct toxicity–efficacy trade-off. Amidi et al. (2022) (27) introduced a machine learning–based forecasting model for ICANS onset, showing high predictive accuracy with CRP, IL-6, and temperature as key variables.

All 10 studies in this section associated increased inflammatory biomarkers or reduced gut resilience with higher risk or severity of ICANS; machine learning models reached sensitivities and specificities typically >70%.

Inflammation, neurotransmitter modulation, and reduced microbial diversity emerged as consistent themes across conditions. Notably, several studies used neuroimaging and cytokine assays to reinforce gut–brain communication pathways.

Across studies, NfL, CRP, IL-6, serum fibrinogen, and immune senescence consistently emerged as predictors for ICANS, while FDG-PET and machine learning tools offered promising stratification models.

The included studies demonstrate considerable heterogeneity in multiple dimensions. This variation encompasses the types of CAR T products administered (e.g., CD19-targeted, BCMA-targeted, and other lineages), the microbiome assessment methods utilized (including 16S rRNA gene sequencing, shotgun metagenomics, and metabolomics approaches), as well as the criteria and scales used for toxicity scoring and neurotoxicity assessment (most notably differences in ICANS grading). Such methodological and clinical diversity across the studies may limit direct comparison and preclude quantitative synthesis, but provides a broad and balanced overview of the current state of research in this emerging area.

### 3.3 Integrative synthesis: mapping the gut–brain–CAR T cell nexus

This review synthesizes emerging evidence across three distinct domains previously considered separately pointing to a possible three-way connection (nexus)—the *Gut–Brain–CAR T Cell Nexus*—through evidence-based integration of findings from 25 human studies across three distinct but biologically overlapping domains. While prior work has focused mainly on individual domains, this synthesis contextualizes potential inter-relationships suggested by currently available studies. This approach, inspired by a Venn convergence model, seeks not merely to collate evidence but to fuse it into a coherent framework for clinical translation (**Table 6**).

**Table 6:** Findings from the nexus: the gut–brain–CAR T cell interface.

Axis	Evidence	Key finding
CAR T + Gut	4 studies	Microbial diversity and antibiotic exposure correlate with response/toxicity
Gut + Brain	11 studies	Microbiota are associated with neurotransmission, inflammation, and neural circuits
CAR T + Brain	10 studies	ICANS associated with immune dysregulation, endothelial stress, and metabolic triggers.
Integrated Nexus	All 3 domains	Microbiota are associated with both CAR T outcomes and ICANS risk via inflammation, metabolites, and immune signaling

Out of 25 studies, in at least 21 (84%) high consistent directionality was observed though data remain preliminary: reduced gut diversity or altered composition is linked to both poorer CAR T efficacy and increased neurotoxicity/brain dysfunction—including heightened systemic inflammation, altered metabolism, and specific neurobehavioral measures. Only 2–3 (12%) studies reported null/ambiguous or inconsistent associations—primarily in the risk score validation or neuroimaging-only substudies.

### 3.4 How integration was achieved

Findings were organized by three domains with partial convergence noted in inflammatory mediators (e.g., CRP, IL-6), metabolic pathways (e.g., SCFAs, glutamine), and neurotoxicity biomarkers (e.g., NfL). The domains are as follows:

1. CAR T Cell Therapy + Gut Microbiota: Gut composition (e.g., diversity, taxa abundance) and function (e.g., metabolite output)are associated with both treatment efficacy and toxicity.
2. Gut Microbiota + Brain: Microbial taxa and their byproducts have been associated with changes in neurotransmitter systems, neuroinflammation, and brain network function across diseases.
3. CAR T Cell Therapy + Brain: CAR T cell–associated neurotoxicity (ICANS) has been associated with endothelial injury, immune activation, and cytokine signaling.

Through structured analysis and mapping of these intersecting threads, the gut microbiota emerged as a potential common factor associated with immune modulation, neurotoxicity, and therapeutic outcomes” OR “gut microbiota showed consistent associations across immune modulation, neurotoxicity, and therapeutic outcomes.

### 3.5 Shared mechanistic threads across domains

Convergent patterns of microbial dysbiosis emerged across both CAR T–associated toxicity and neuropsychiatric disease states, characterized predominantly by diminished alpha diversity and marked depletion of butyrate-producing commensals, including Faecalibacterium and Coprococcus species (2, 5, 10, 19). Smith et al. (10) and Hu et al. (12) demonstrated that compromised microbial diversity anteceded the clinical manifestation of immune effector cell-associated neurotoxicity syndrome (ICANS) and portended inferior therapeutic responses to CAR T cellular immunotherapy. Strikingly parallel findings were documented by Procházková et al. (18) and Valles-Colomer et al. (19) in patient cohorts with anorexia nervosa and major depressive disorder, respectively, wherein comparable microbial perturbations correlated with neuropsychiatric symptom severity. These observations substantiate the hypothesis that intestinal microbiota composition is associated with neuroimmune homeostasis across diagnostic categories.

The mechanistic nexus between enteric dysbiosis and central nervous system pathology was further elucidated through characterization of systemic inflammatory mediators. Larue et al. (25) and De Matteis et al. (31) identified elevated circulating concentrations of C-reactive protein (CRP), interleukin-6 (IL-6), and tumor necrosis factor-alpha (TNF-α) as prognostic biomarkers for ICANS severity and temporal onset. These inflammatory signatures recapitulated findings from neurodegenerative disease cohorts, wherein Lin et al. (15) and Cao et al. (17) established that gut-derived inflammatory mediators disrupted neural network integrity in Parkinson’s disease and contributed to post-stroke aphasic deficits. Collectively, these data implicate chronic, low-grade systemic inflammation—originating from intestinal barrier dysfunction and microbial dysbiosis—as a unifying pathophysiological mechanism underlying both peripheral immunological perturbations and central nervous system dysregulation.

The gut microbiota’s capacity to modulate neurotransmitter biosynthesis and availability represents an additional mechanistic convergence point. Multiple investigations have demonstrated that microbial-derived metabolites, including short-chain fatty acids (SCFAs) and neurotransmitter precursors such as tryptophan, serotonin, and dopamine, are critically involved in both psychiatric pathophysiology and CAR T-associated neurotoxicity (13, 20, 23). Jalota et al. (13) identified profound glutamine depletion as a metabolic harbinger of ICANS onset, while Wan et al. (20) and Lin et al. (23) delineated perturbations in serotonergic and kynurenine metabolic pathways associated with attention-deficit/hyperactivity disorder and major depressive disorder, respectively—both conditions demonstrating concurrent alterations in intestinal microbial composition. These findings underscore the microbiome’s role as a remote regulator of central neurochemical homeostasis through production of neuroactive metabolites capable of traversing the gut–brain axis.

Disruption of blood–brain barrier (BBB) integrity constituted a cardinal feature of ICANS pathogenesis. Gust et al. (8) documented extensive BBB compromise accompanied by systemic endothelial activation following administration of CD19-directed CAR T cells. These observations align with emerging evidence from gut-brain axis research demonstrating that depletion of microbial-derived SCFAs—particularly butyrate—in conjunction with systemic immune activation, precipitates barrier dysfunction across both intestinal epithelium and cerebrovascular endothelium (8, 14, 18, 21). The loss of butyrate, a metabolite with established capacity to reinforce tight junction protein expression and barrier integrity, may therefore represent a shared vulnerability predisposing individuals to both systemic inflammatory propagation and neurotoxic sequelae.

Pre-existing metabolic and immunological dysregulation emerged as a critical determinant of clinical outcomes following CAR T therapy and across neuropsychiatric disease trajectories. Baseline phenotypes characterized by elevated CD8+ T cell senescence, diminished microbial butyrate biosynthetic capacity, and aberrant tryptophan catabolism were associated with increased ICANS incidence and therapeutic failure following CAR T infusion (5, 10, 31). Analogous metabolic perturbations—including dysregulated kynurenine-to-serotonin ratios and cerebral glucose hypometabolism—have been implicated in the pathogenesis of psychiatric disorders and neurodegenerative conditions (2, 19, 20, 23). These parallel observations suggest that an individual’s baseline metabolic and microbial landscape may function as a prognostic determinant of neuroimmune resilience and therapeutic responsiveness across disparate clinical contexts.

## 4 Discussion

### 4.1 Summary of key findings

This review does not confirm a mechanistic ‘axis’ but instead synthesizes evidence from three domains (CAR T–gut, gut–brain, and CAR T–neuro). Points of convergence suggest shared mediators such as CRP, IL-6, SCFAs, and NfL. These findings represent a preliminary convergence model requiring validation. Therefore, we frame this work as a tri-domain convergence model rather than a fully integrated axis. Drawing from 25 selected human studies, we synthesized findings across three mechanistic interfaces: (1) CAR T cell therapy and gut microbiota, (2) gut microbiota and the brain, and (3) CAR T cell therapy and neurotoxicity. Each of these domains has independently garnered scientific attention in recent years, yet no prior work has linked them through a unified biological model.

The evidence reviewed indicates that the gut microbiome may play an upstream regulatory role, with its influence radiating outward through a triad of interconnected physiological systems. Across studies, alterations in microbial diversity (e.g., alpha diversity, SCFA-producing taxa), metabolite production (e.g., glutamine, serotonin, butyrate), and systemic inflammatory tone (e.g., CRP, IL-6, kynurenine levels) were repeatedly shown to impact both CAR T cell efficacy and neurotoxicity, as well as cognitive, psychiatric, and neurodegenerative outcomes.

Importantly, this synthesis allowed us to suggest a possibleconceptual framework: the tripartite gut–brain–CAR T cell nexus. This framework links traditionally siloed research domains—oncologic immunotherapy, microbiome science, and neuropsychiatric medicine—through shared biological hallmarks. These include:

- Predictive biomarkers [e.g., serum NfL, CRP, microbial richness (Shannon diversity index, Chao1 estimator)]
- Immune-metabolic pathways (e.g., kynurenine-tryptophan-serotonin metabolism, SCFA signaling)
- Pathophysiological interfaces (e.g., blood–brain barrier permeability, neuroimmune activation, senescent T cell burden)

### 4.2 Contextualization within existing literature

In the CAR T–gut microbiota interface, we found that antibiotic-induced dysbiosis, particularly involving agents like piperacillin-tazobactam or meropenem, was consistently associated with decreased microbial diversity, worsened progression-free survival, and elevated incidence of ICANS (5, 10, 12). Notably, emerging observational data suggest that non–anti-anaerobic regimens may be less disruptive to the microbiome than broad-spectrum anti-anaerobic agents, reinforcing the importance of antibiotic stewardship in CAR T recipients (5, 10). *Akkermansia*, *Bacteroides*, and *Faecalibacterium* emerged as beneficial taxa, while butyrate producers were linked to improved CAR T responses (5, 10, 12). These findings echo prior work in checkpoint inhibitor therapy, where the gut microbiome has also shown correlations with immunotherapy efficacy and immune-related adverse events (11).

In the gut–brain axis, the gut microbiota’s association with neurotransmission, inflammation, and network topology was substantiated across a range of conditions. Elevated CRP, IL-6, kynurenine, and decreased serotonin or SCFA levels were common biochemical correlates (15, 16, 18–20, 23). Notably, alterations in gut microbial are associated with GABAnergic and dopaminergic signaling— reported in conditions such as ADHD, depression, and schizophrenia may share features with neurotransmitter changes observed in CAR T-related neurotoxicity, suggesting a possible mechanistic link between immune activation and cognitive effects (16, 19, 20, 23).

On the CAR T–brain side, ICANS was driven by endothelial activation, BBB leakage, and cytokine surges, with neurofilament light chain (NfL), serum fibrinogen, and EASIX scores emerging as early predictors (8, 24, 25, 29). Imaging findings such as orbitofrontal hypometabolism on FDG-PET further validated the clinical neurotoxicity landscape (26).

This study integrates these findings through a Venn model—where each domain overlaps via shared mediators (e.g., CRP, IL-6, microbial taxa, glutamine, NfL)—positioning the gut microbiota as the initiating hub that are associated with both immune homeostasis and neuroinflammatory vulnerability during CAR T therapy.

### 4.3 Implications for clinical practice and translational research

These insights open new translational frontiers. Microbiome profiling and modulation remain exploratory. While signals from observational studies are encouraging, interventions such as probiotics, prebiotics, or FMT should be considered hypotheses-generating only, pending validation in prospective interventional trials. The observation of antibiotic exposure, NfL, SCFA levels, and microbial richness support integrating gut monitoring into CAR T pre-conditioning protocols. Second, leveraging neuroprotective microbial pathways—through targeted probiotic therapies, prebiotics, or even fecal microbiota transplantation (FMT)—may offer non-immunosuppressive strategies to prevent ICANS while preserving CAR T efficacy. Importantly, microbiome interventions such as probiotics and fecal microbiota transplantation (FMT) are currently considered investigational in the context of CAR T cell therapy. There is no established evidence supporting their routine use in clinical practice, and their efficacy and safety require confirmation in well-designed clinical trials.

From a policy perspective, findings argue for restrictive antibiotic stewardship in CAR T patients, not only to reduce opportunistic infections, but to preserve microbial symbiosis crucial to treatment response and neurological safety. Lastly, our synthesis suggests that neurotoxicity is not merely an isolated side effect, but a systems-level phenomenon are associated with host–microbiota–immune–brain interactions, necessitating new avenues for investigation in ICANS management.

### 4.4 Strength of evidence

The application of the GRADE framework provided insights into the certainty of evidence across different factors. The details are summarized in **Table 7**.

**Table 7:** Summary of findings table with GRADE.

Outcome	No. of studies (design)	Main finding	Representative quantitative examples	Certainty (GRADE)	Reasons for downgrading
CAR T efficacy *vs* gut microbiome (CR/PFS/OS)	3 observational cohorts	Higher microbial diversity and enrichment of Akkermansia and Bacteroides linked to improved response/survival. Broad-spectrum antibiotics before infusion associated with worse PFS/OS.	Smith 2022: Any antibiotics → PFS HR 1.83 (95% CI 1.03–3.27); OS HR 3.37 (95% CI 1.77–6.44). Stein-Thoeringer 2023^3^: Pre-infusion antibiotics significantly worsened outcomes (HR ~2.0, 95% CI reported in Extended Data). Hu 2022: Specific taxa associated with CAR T response and CRS severity (effect sizes reported).	⊕⊕◯◯ Low	Risk of bias (observational, potential confounding by indication); Imprecision (few cohorts, heterogeneous CAR T products and endpoints).
ICANS prediction — inflammatory/injury biomarkers	6 observational studies	Elevated inflammatory (CRP, IL-6), endothelial (fibrinogen, LDH, EASIX), and neuronal injury (NfL) markers consistently associated with ICANS risk/severity.	Larue 2024: NfL >58 pg/mL → OR 4.3 (95% CI 1.3–13.7) for ICANS 2–4. de Boer 2023: EASIX-family AUC 0.61–0.62 for ICANS ≥2 (weak predictive value). Holtzman 2021: Elevated fibrinogen and LDH predicted ICANS (OR NR). Amidi 2022: ML model using CRP, IL-6, temperature achieved AUROC ~0.96 (CI NR). Gust 2017^6^: Endothelial activation linked to severe ICANS. De Matteis 2023: Senescent CD8+, CRP, NfL associated with ICANS.	⊕⊕⊕◯ Moderate	Risk of bias (observational, variable confounder adjustment); Inconsistency (different biomarkers, thresholds, and timing across studies).
ICANS risk stratification scores	2 observational studies	Risk scores show limited–moderate predictive performance for ICANS.	de Boer 2023: EASIX-family AUC ~0.61 for ICANS ≥2 (weak predictive value). Amidi 2022: Integrated ML model AUROC ~0.96 using clinical + biomarker features.	⊕⊕◯◯ Low	Risk of bias (retrospective, potential overfitting); Imprecision (only 2 studies, inconsistent performance, no CI).
ICANS neuroimaging correlates	1 observational study	Frontal/orbitofrontal hypometabolism on FDG-PET associated with ICANS, distinct from CRS-only patterns.	Morbelli 2023: ICANS patients showed orbitofrontal hypometabolism compared with parietal/temporal changes in CRS-only; quantitative effect sizes not reported.	⊕⊕◯◯ Low	Risk of bias (ancillary study, small sample); Imprecision (single study, no standardized effect metrics).
ICANS clinical phenotype	2 observational studies	Early neurological signs may precede formal ICANS diagnosis; ICANS presence does not necessarily impair long-term CAR T efficacy.	Sales 2024: Early handwriting/tremor changes tracked with ICANS onset (descriptive). Ma 2024: ICANS did not affect OS, CR, or LFS in pediatric B-ALL cohort.	⊕⊕◯◯ Low	Risk of bias (small, single-center studies); Imprecision (limited quantitative data, no standardized assessments).
ICANS predictors — metabolomics	1 observational study	Pre-treatment metabolite depletion (glutamine, hydroxyproline) associated with earlier and more severe ICANS/CRS.	Jalota 2023: Significant glutamine reduction (>25%) in ICANS+ *vs* ICANS– patients; effect size NR.	⊕◯◯◯ Very Low	Risk of bias (single-center exploratory); Inconsistency (no replication); Imprecision (small n, no CI).
Gut microbiota and neurological/psychiatric outcomes (non-CAR T)	11 observational studies	Reduced microbial diversity, altered SCFA-producing taxa, and neurotransmitter pathway dysregulation associated with neurological and psychiatric conditions.	Lin 2019 (PD): Dysbiosis correlated with ↑IL-6 and motor severity. Wu 2024 (schizophrenia): ↓butyrate producers, ↑inflammation, altered brain connectivity. Valles-Colomer 2019: Faecalibacterium, Coprococcus abundance protective for QoL/depression. Ning 2022: MR: Ruminococcus ↑ALS risk; serotonin protective in PD.	⊕⊕⊕◯ Moderate	Risk of bias (mostly observational); Inconsistency (heterogeneous populations, methods, and outcomes).
Therapeutic interventions for ICANS	1 observational study	Early corticosteroid use may reduce CRS without worsening ICANS.	Lakomy 2023: Early steroids decreased CRS severity without increasing ICANS incidence.	⊕⊕◯◯ Low	Risk of bias (retrospective, confounding by indication); Imprecision (single cohort, no randomized data).

### 4.5 Limitations

Most included studies were observational with heterogeneous populations, CAR T products, microbiome assays, and sampling windows, which limited quantitative pooling and introduced inconsistency. Several studies lacked standardized ICANS assessment windows or comprehensive confounder adjustment. Most included studies were observational, often with small sample sizes and heterogeneous endpoints, leading us to downgrade certainty. We caution that biomarkers such as NfL, CRP, and IL-6 show early predictive associations with ICANS risk, though thresholds and reproducibility vary across studies. These limitations reduce the certainty of evidence, as reflected in GRADE assessments.

**Figure 2:**
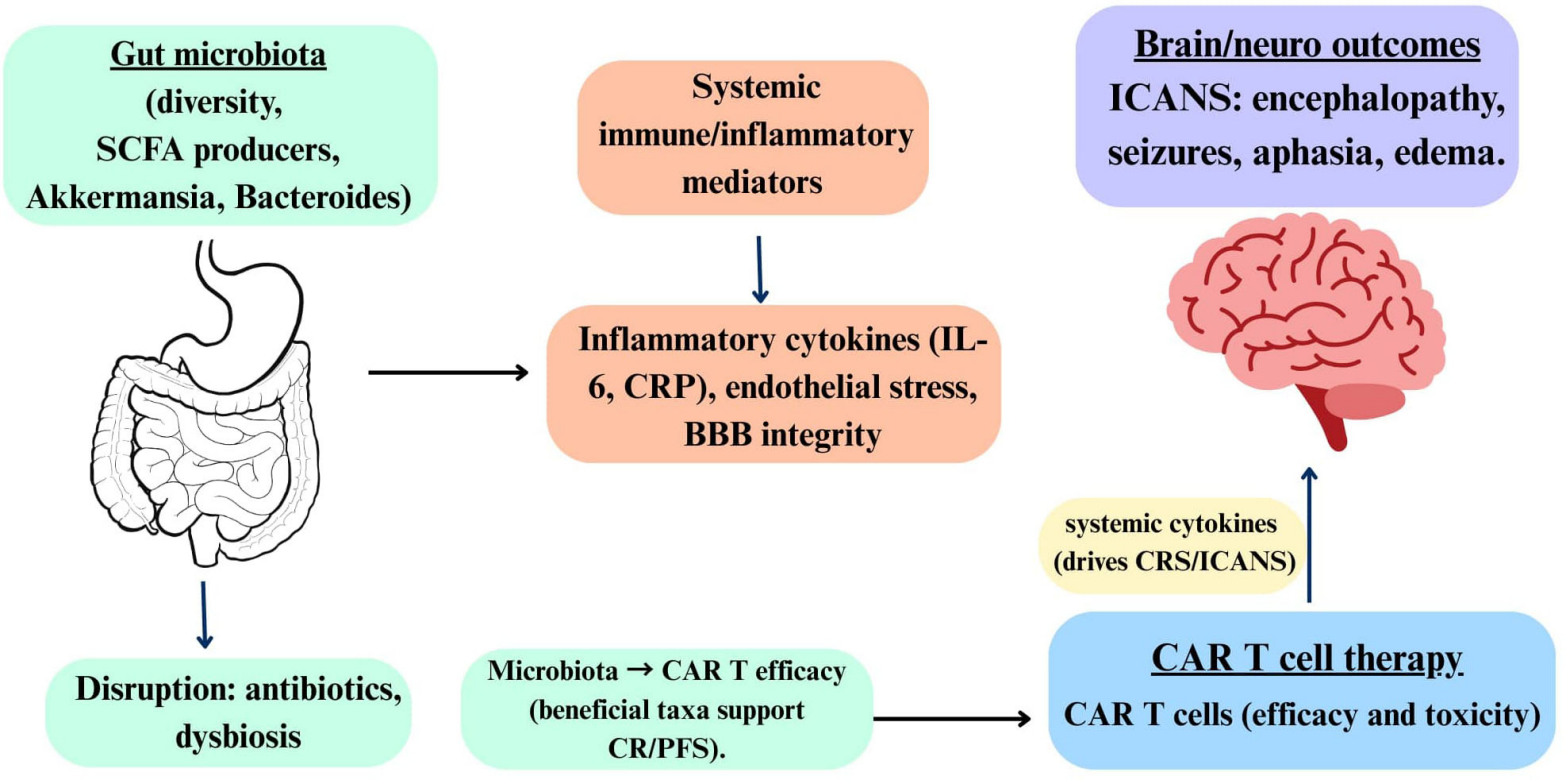
Hypothesized associations between gut microbiota, systemic inflammation, and CAR T-cell therapy outcomes. This framework synthesizes correlational evidence from 25 studies across three domains: gut microbiota and CAR T-cell therapy (n=4), gut microbiota and brain outcomes (n=11), and CAR T-cell neurotoxicity (n=10). Gut microbiota characteristics (diversity, SCFA-producing taxa) show associations with inflammatory mediators (IL-6, CRP, endothelial stress), which correlate with ICANS severity and CAR T-cell efficacy. Microbiome disruption through antibiotics or dysbiosis is associated with altered outcomes. Visual Key: • Solid lines indicate associations supported by ≥2 studies.• Dashed lines represent hypothetical pathways (<2 studies) or untested connections. • Bi-directional arrows indicate associations without established causal direction. • The dashed framework border emphasizes that all relationships are correlative, not causal, and require prospective validation. CRITICAL DISCLAIMER: All connecting lines and arrows represent observed ASSOCIATIONS, not proven causal pathways. Directionality of relationships has not been established. This is a hypothesis-generating conceptual model requiring prospective mechanistic validation. BBB, blood-brain barrier; CAR, chimeric antigen receptor; CR, complete remission; CRP, C-reactive protein; CRS, cytokine release syndrome; ICANS, immune effector cell-associated neurotoxicity syndrome; IL-6, interleukin-6; PFS, progression-free survival; SCFA, short-chain fatty acid.

**Figure 3:**
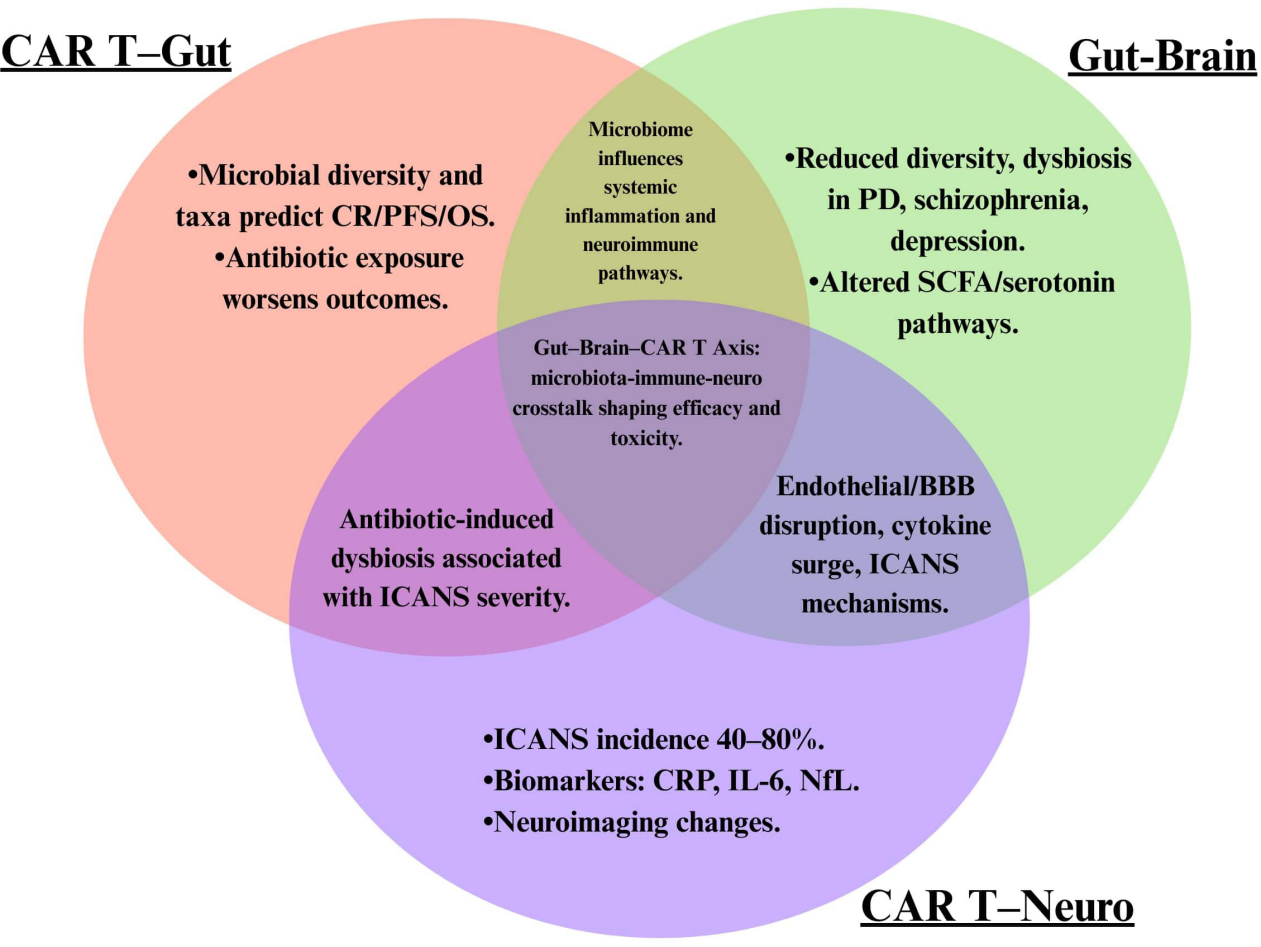
Gut-Brain-CAR T Association Nexus: Overlapping Evidence Domains This Venn diagram illustrates the current evidence base across three research domains: CAR T-gut microbiota interactions (purple), gut-brain axis relationships (green), and CAR T neurotoxicity (tan). Overlapping regions represent areas where multiple domains converge, with the central area highlighting the conceptual integration of all three axes. All statements reflect associations reported in the literature, not causal relationships. ICANS, immune effector cell-associated neurotoxicity syndrome; CR, complete remission; PFS, progression-free survival; OS, overall survival; PD, Parkinson’s disease; SCFA, short-chain fatty acids; CRP, C-reactive protein; IL-6, interleukin-6; NfL, neurofilament light chain; BBB, blood-brain barrier. CRITICAL DISCLAIMER: Overlapping regions represent shared CORRELATES and biomarkers identified across studies, NOT proven mechanistic connections. The positioning of mediators (CRP, IL-6, SCFA, NfL) in overlapping zones indicates they were reported in studies from multiple domains but does not establish causal links between domains. Important: This diagram depicts associations and convergence patterns, not a validated biological mechanism.

An important limitation concerns the visual presentation of our conceptual framework. **Figures 2** and **3** use connecting lines and arrows to illustrate hypothesized relationships and evidence convergence. While we have included disclaimers in the figure legends, we acknowledge that the graphical format may inadvertently suggest causality to readers. We emphasize that all relationships shown are correlative and observational. Directionality of effects has not been established through experimental manipulation or prospective interventional studies. The frameworks should be interpreted as hypothesis-generating models rather than established biological mechanisms. Readers should refer to the accompanying legends and this limitation statement for appropriate interpretation.

### 4.6 Future directions

This review establishes a strong rationale for:

1. Prospective, multicenter trials evaluating microbiome-guided ICANS prevention strategies (e.g., FMT, SCFA supplementation, restrictive antibiotic stewardship).
2. Development of multi-domain risk prediction tools, integrating gut taxa, cytokines (especially IL-6), and neurological biomarkers (e.g., NfL, MRI changes).
3. Mechanistic studies linking microbial-derived metabolites to BBB permeability and neurotoxicity in CAR T models.

Multi-omics, single-cell sequencing, and AI-driven biomarker integration will be critical to deciphering and personalizing the gut–brain–CAR T nexus. Future research should directly investigate mechanistic bridges across these domains—for example, microbiota-derived metabolites modulating BBB permeability during CAR T therapy—to determine whether the concept of a true Gut–Brain–CAR T nexus is supported.

## 5 Conclusion

In conclusion, this review presents a novel and comprehensive, human-based synthesis of the Gut–Brain–CAR T Cell Nexus, highlighting the gut microbiota as a biological keystone linking immune efficacy and neurotoxicity in CAR T therapy. The convergence of immune, microbial, and neural pathways not only underscores the need for microbiome-aware oncology but also opens a new paradigm for neurotoxicity prevention, patient stratification, and precision immunotherapy.

## Data Availability

The original contributions presented in the study are included in the article/**Supplementary Material**. Further inquiries can be directed to the corresponding author.

## Glossary

ADHD: Attention-Deficit/Hyperactivity Disorder
ASCO: American Society of Clinical Oncology
ASH: American Society of Hematology
AUROC: Area Under the Receiver Operating Characteristic
AUC: Area Under the Curve
BBB: Blood–Brain Barrier
B-ALL: B-cell Acute Lymphoblastic Leukemia
CAR T: Chimeric Antigen Receptor T-cell
CI: Confidence Interval
CR: Complete Remission
CRP: C-reactive protein
CRS: Cytokine-Release Syndrome
EBMT: European Society for Blood and Marrow Transplantation
EASIX: Endothelial Activation and Stress Index
FDG-PET: 18F-Fluorodeoxyglucose Positron Emission Tomography
FMT: Fecal (Faecal) Microbiota Transplantation
GABA: Gamma-Aminobutyric Acid
GRADE: Grading of Recommendations, Assessment, Development and Evaluations
HR: Hazard Ratio
ICANS: Immune Effector Cell–Associated Neurotoxicity Syndrome
IL-6: Interleukin-6
JITC: *Journal for ImmunoTherapy of Cancer* (journal name
LDH: Lactate Dehydrogenase
LFS: Leukemia-Free Survival
MDD: Major Depressive Disorder
MR: Mendelian Randomization
MRD: Measurable Residual Disease
NfL: Neurofilament Light Chain
NOS: Newcastle–Ottawa Scale
OR: Odds Ratio
OS: Overall Survival
PD: Parkinson’s Disease
PET: Positron Emission Tomography
PICO: Population, Intervention, Comparator, Outcome
PFS: Progression-Free Survival
PRISMA: Preferred Reporting Items for Systematic Reviews and Meta-Analyses
PROSPERO: International Prospective Register of Systematic Reviews
rRNA (16S rRNA): Ribosomal Ribonucleic Acid (16S region)
SCFA: Short-Chain Fatty Acid
