# Supplementary material for: The gut microbiota–brain–CAR T cell axis: a systematic review of gut microbiome modulation and its impact on neurological complications and treatment responses in CAR T cell therapy
Source: Front Immunol. 2026 Jan 5;16:1703146. doi: 10.3389/fimmu.2025.1703146 (PMC12813054; doi:10.3389/fimmu.2025.1703146)
Supplement: Supplementary file 1 [file Table1.docx]

**Supplementary Table S1.** Search strategies of the databases searched, along with the number of results

| **Database** | **Search Strategy** | **Number** |
| --- | --- | --- |
| PubMed | ("CAR T" OR "CAR-T" OR "chimeric antigen receptor") AND (microbiome OR microbiota OR microbiology OR bacteria OR "gut flora" OR "intestinal flora" OR "gut" OR "intestinal" OR "commensal" OR "dysbiosis" OR antibiotics OR probiotics OR prebiotics OR "fecal transplant" OR "fecal microbiota transplantation" OR FMT) | 1415 |
| Embase | ('chimeric antigen receptor t cell therapy'/exp OR 'CAR T cell':ab,ti OR 'CAR T':ab,ti OR 'chimeric antigen receptor':ab,ti OR 'adoptive cell therapy':ab,ti)  AND  ('gut microbiota'/exp OR 'gut microbiome':ab,ti OR microbiome:ab,ti OR microbiota:ab,ti OR 'intestinal flora'/exp OR 'intestinal flora':ab,ti OR dysbiosis:ab,ti OR 'fecal microbiota transplantation'/exp OR 'fecal transplant':ab,ti OR probiotics:ab,ti OR prebiotics:ab,ti OR antibiotics:ab,ti) | 456 |
| Scopus | TITLE-ABS-KEY(("CAR T" OR "chimeric antigen receptor"))  AND  TITLE-ABS-KEY(("microbiome" OR "microbiota" OR "gut microbiome" OR "gut microbiota" OR antibiotics OR probiotics OR dysbiosis OR "fecal microbiota transplantation" OR "intestinal flora")) | 636 |
